# How many days are needed to estimate wrist-worn accelerometry-assessed physical activity during the second trimester in pregnancy?

**DOI:** 10.1371/journal.pone.0211442

**Published:** 2019-06-27

**Authors:** Shana Ginar da Silva, Kelly R. Evenson, Ulf Ekelund, Inácio Crochemore Mohsam da Silva, Marlos Rodrigues Domingues, Bruna Gonçalves Cordeiro da Silva, Márcio de Almeida Mendes, Gloria Isabel Niño Cruz, Pedro Curi Hallal

**Affiliations:** 1 PostGraduate Program in Epidemiology, Federal University of Pelotas, Pelotas, RS, Brazil; 2 Medical School, Federal University of Fronteira Sul–Passo Fundo, Passo Fundo, RS, Brazil; 3 Department of Epidemiology, Gillings School of Global Public Health, University of North Carolina–Chapel Hill, Chapel Hill, North Carolina, United States of America; 4 Department of Sports Medicine, Norwegian School of Sport Sciences, Oslo, Norway; 5 PostGraduate Program in Physical Education, Federal University of Pelotas, Pelotas, RS, Brazil; University of Maiduguri College of Medical Sciences, NIGERIA

## Abstract

**Background:**

Objective methods to measure physical activity (PA) can lead to better cross-cultural comparisons, monitoring temporal PA trends, and measuring the effect of interventions. However, when applying this technology in field-work, the accelerometer data processing is prone to methodological issues. One of the most challenging issues relates to standardizing total wear time to provide reliable data across participants. It is generally accepted that at least 4 complete days of accelerometer wear represent a week for adults. It is not known if this same assumption holds true for pregnant women.

**Aim:**

We assessed the optimal number of days needed to obtain reliable estimates of overall PA and moderate-to-vigorous physical activity (MVPA) during the 2nd trimester in pregnancy using a raw triaxial wrist-worn accelerometer.

**Methods:**

Cross-sectional analyses were carried out in the antenatal wave of the 2015 Pelotas (Brazil) Birth Cohort Study. Participants wore the wrist ActiGraph wGT3X-BT accelerometer for seven consecutive days. The daily average acceleration, which indicated overall PA, was measured as milli-*g* (m*g*), and time spent in MVPA (minutes/day) was analyzed in 5-minute bouts. ANOVA and Kruskal-Wallis tests were used to compare variability across days of the week. Bland-Altman plots and the Spearman-Brown Prophecy Formula were applied to determine the reliability coefficient associated with one to seven days of measurement.

**Results:**

Among 2,082 pregnant women who wore the accelerometer for seven complete days, overall and MVPA were lower on Sundays compared to other days of the week. Reliability of > = 0.80 to evaluate overall PA was reached with at least three monitoring days, whereas seven days were needed to estimate reliable measures of MVPA.

**Conclusions:**

Our findings indicate that obtaining one week of accelerometry in adults is appropriate for pregnant women, particularly to obtain differences on weekend days and reliably estimate overall PA and MVPA.

## Introduction

Objective methods to measure physical activity (PA), such as accelerometers, have become widely used over the years since it provides more accurate parameters to assess patterns of PA in free-living conditions [1]. Accelerometry-based PA assessment can lead to better cross-cultural comparisons, monitoring temporal PA trends and measuring the effect of interventions [2]. However, when applying this technology to field work, the accelerometer data processing is prone to methodological issues with important implications that can affect data quality [3,4]. One of the most challenging issues relates to standardizing total wear time to provide reliable data across participants [5–8].

Studies have been carried out in children [9], young [10,11], and adult populations [10,12,13] focused on the number of monitoring days necessary to represent habitual PA behavior. These studies suggested a large variability in the number of days required to obtain reliable measures of PA ranging from 2 to 9 days. Also, the number of required days varied according to the intensity of physical activities, often grouped as sedentary behavior, and light, moderate, and vigorous intensity [10, 12,13]. Other factors that can influence the monitoring time-frame are the type of accelerometer used and placement of the device (e.g., wrist, thigh, or hip) [6–8].

A growing interest in PA during pregnancy has emerged given the potential positive effects of PA on maternal-child health [14]. However, there are currently few studies which have used accelerometers to measure PA during pregnancy [15,16]. Moreover, studies to determine a suitable monitoring time-frame to accurately measure PA behavior has been performed in young to middle-aged adults [10–13], and no data appear available among pregnant women.

Recently, our research team published a paper that assessed the correlates of accelerometer assessed physical activity in pregnancy [17]. The criterion of four days of measurement to represent a week was applied, based on preliminary analyses. Subsequently, we performed methodological analysis to explore in depth the reliability of objectively measured PA from one to seven monitoring days in pregnant women. Therefore, the purpose of the present study was to examine the optimal number of days needed to obtain reliable estimates of overall PA and MVPA during the 2nd trimester in pregnancy using a raw data from a triaxial wrist-worn accelerometer in a population-based study in southern Brazil. In addition, we measured the variability in PA across days of the week.

## Materials and methods

### Design and participants

We conducted cross-sectional analyses based on the antenatal wave of the 2015 Pelotas (Brazil) Birth Cohort Study. Participants with an expected delivery date from January 1^st^ 2015 to December 31^st^ 2015 were eligible for the cohort and recruited from all public and private health facilities offering antenatal care in the city of Pelotas. Accelerometry data was collected between weeks 16 and 24 of gestation. Details regarding the study have been previously described elsewhere [18]. Ethical approval was obtained from the Ethics Committee of the Physical Education School—Federal University of Pelotas, in accordance with official letter numbered 522/064, approved the study. All participants signed a written informed consent prior to participation.

### Measurements

The accelerometer used was the ActiGraph wGT3X-BT models (ActiGraph, Pensacola, FL, USA). These devices were lightweight (27 g) and compact (3.8 × 3.7 × 1.8 cm), allowing measurement of body movements over three orthogonal axes (vertical (Y), horizontal right-left (X), and horizontal front-back axis (Z)) within an acceleration dynamic range of ± 8g [19]. Participants wore the accelerometer on their non-dominant wrist (dorsally midway between the radial and ulnar styloid processes) during 24 hours for seven consecutive days. In order to define the non-dominant wrist, pregnant women were asked about which hand they usually used to write or perform most daily activities. The accelerometer was programmed to collect raw acceleration at 60 Hz and three-dimensional raw data was expressed in gravitational equivalent units called milli-gravity (*mg*, where 1000*mg* = 1*g* = 9.81 m/s^2^).

### Data reduction

Accelerometers were programmed and data downloaded using ActiLife software, version 6.11.7. Accelerometer raw data analyses were performed in R-package GGIR [20]. Two parameters were used to consider valid data for the analyses: calibration error <0.02*g* and seven full days of measurement. The minimum required wearing time to be considered a valid day was 16 hours per day, based on the GGIR recommendation [20]. Euclidian Norm Minus One (ENMO) was used to summarize three-dimensional raw data (from axes x, y, and z) into a single-dimensional signal vector magnitude ($SVM=\sum |\sqrt{x^{2}{+y}^{2}+z^{2}}-1g|$) [19]. Data were further summarized when calculating the average values per 5-second epochs. The summary measures used were (a) overall PA (expressed in m*g*), based on the average SVM per day and (b) average time spent in MVPA per day with 5-minute bouts criterion (expressed in minutes). MVPA was defined as SVM records above 100m*g* [21,22], while bouts were defined as consecutive periods in which participants spent at least 80% of the time in activities with intensity equal or higher the MVPA threshold.

### Statistical analysis

Sample descriptions are presented in relative (%) and absolute frequencies (N). Overall PA was expressed as a mean and standard deviation (SD), while MVPA was presented as a mean, SD, median, and interquartile range (25th and 75th percentiles). Overall PA and MVPA were checked graphically using a histogram and by the mean, median, skewness, and kurtosis. Because of positive skewness and in order to meet the assumptions of the symmetry required for intraclass correlations, MVPA was log-transformed for the analyses, while total PA met the assumptions. Analysis of variance (ANOVA) and Kruskal-Wallis non-parametric tests were used to compare whether PA varied significantly across days of the week. If an overall significant F level was shown, post-hoc tests (Bonferroni pairwise comparisons) were used to assess differences between weekdays. The number of days required to reliably estimate habitual PA (overall PA and MVPA) was assessed using the Spearman-Brown formula. A modified version of the Spearman-Brown calculation determined the intraclass reliability coefficient associated with 1 to 7 days of measurement. The standard typically used for acceptable reliability was an intraclass correlation coefficient of > = 0.80 [23]. We also assessed agreement based on the visual inspection of the Bland-Altman plots.

In order to explore differences in results by sociodemographic characteristics and body mass, we stratified the analysis by maternal age (<20, 20–29, 30–39, ≥40), skin color (white, black, brown/yellow/indigenous), socioeconomic position (based on asset index [24] and later categorized into quintiles), paid job during pregnancy (yes/no), and pre-pregnancy body mass index (BMI) (calculated by dividing weight by height squared (kg/m^2^) with cutoffs defined according to the World Health Organization [25]). All analyses were performed using Stata version 12.1 (StataCorp, College Station, TX, USA). Statistical significance was set at *α* < 0.05.

## Results

From 2,463 pregnant women with accelerometry data, 2,082 adhered to the research protocol and wore the accelerometer for seven consecutive days. A high proportion of the sample was aged 20–29 (49.5%), had white skin color (73.3%), did not have a paid job during pregnancy (50.1%), had a normal pre-pregnancy BMI (48.8%) and belonged to the top quintile for socio-economic position (Table 1).

**Table 1:** Characteristics of participants that wore accelerometer for seven consecutive days. The 2015 Pelotas (Brazil) birth cohort study.

	n	%
**Maternal age (years)**		
<20	277	13.3
20–29	1,029	49.5
30–39	722	34.7
≥ 40	52	2.5
**Skin color**		
White	1,523	73.3
Black	255	12.3
Brown/yellow/indigenous	299	14.4
**SES (quintiles)**		
Q1(poorest)	243	14.7
Q2	327	19.8
Q3	358	21.7
Q4	359	21.7
Q5 (wealthiest)	366	22.1
**Paid job during pregnancy**		
No	1,042	50.1
Yes	1,037	49.9
**Pre-pregnancy BMI (kg/m**^**2**^**)**		
Underweight	61	3.3
Normal	913	48.8
Overweight	536	28.7
Obese	360	19.3

SES: socioeconomic position; BMI: body mass index.

Mean overall PA (m*g*) and time spent in MVPA (minutes/day) was was lower on Sunday (25.6 m*g* and 8.6 minutes/day, respectively) compared to all other days (Table 2). Pregnant women were more physically active on weekdays and Saturday (p<0.001) for overall PA and on weekdays (p<0.001) for MVPA.

**Table 2:** Daily duration (*mg* and minutes) of overall physical activity and moderate to vigorous physical activity.

	Overall PA (*mg)*			MVPA (minutes/day)					
	Mean	SD	*p*^*a*^	Mean	SD	*p*^*b*^	Median	Interquartile range	*p* ^b^
			**<0.001**			**<0.001**			**<0.001**
Monday	28,0	8,9		15,5	21,5		7,5	0–22	
Tuesday	28,2	8,8		15,2	20,9		6,9	0–22	
Wednesday	28,2	9,2		15,2	21,0		7,3	0–21	
Thursday	28,4	8,7		16,5	22,5		8,8	0–24	
Friday	28,6	9,0		16,0	21,8		8,3	0–23	
Saturday	28,3	8,7		12,5^ǂ^	19,0		5,2	0–17	
Sunday	25,4^ǂ^	7,9		8,6^ǂ^	15,5		0	0–11	

^a^ANOVA

^b^Kruskal-Wallis’ non-parametric test

^ǂ^Bonferroni’s test

MVPA: moderate-to-vigorous physical activity. PA: physical activity. SD: standard deviation

**Fig 1:**
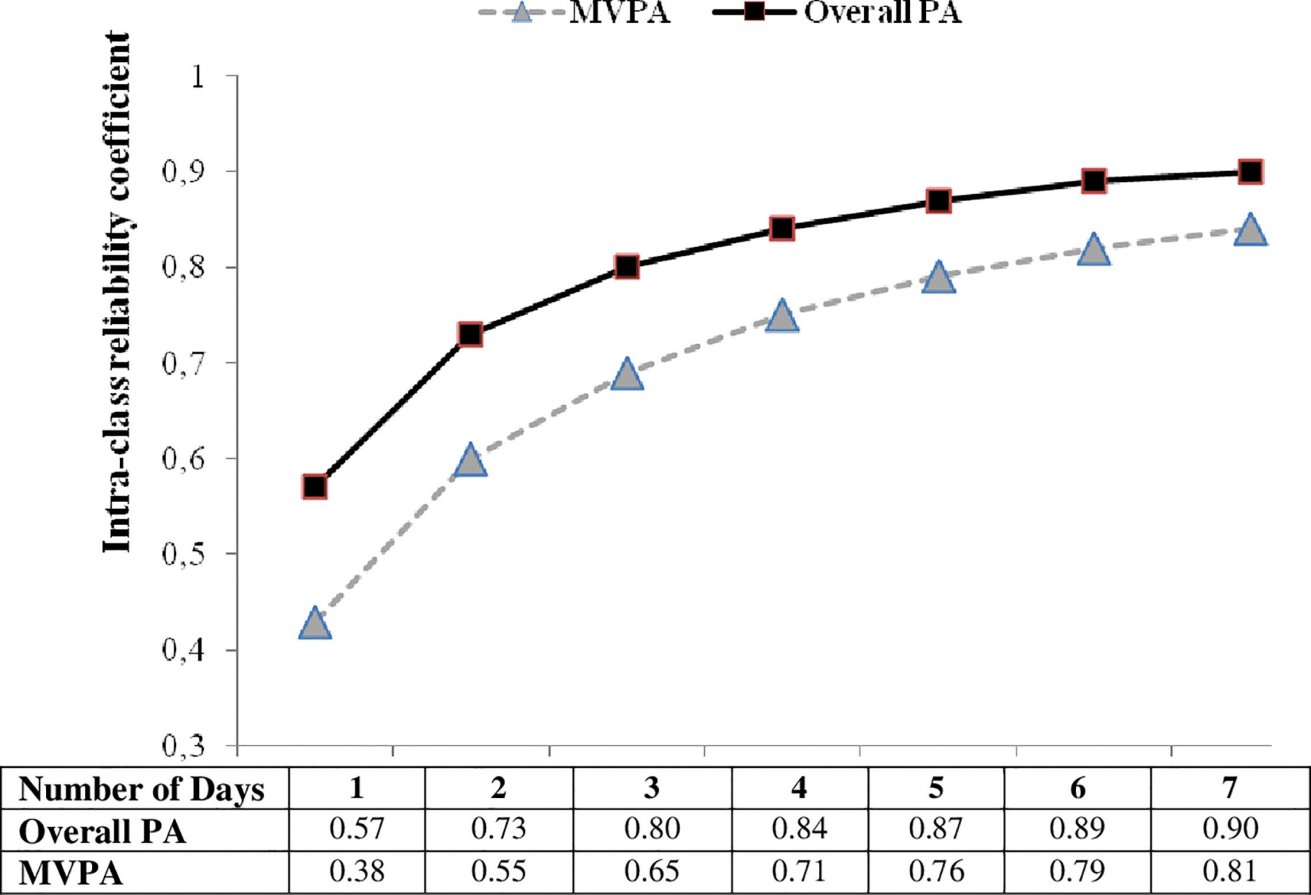
Intraclass reliability coefficient for the number of days monitoring overall PA and MVPA.

Estimates of the number of days needed to obtain reliable measures of habitual PA are presented in Fig 1.

For overall PA, at least three days of the week was the minimum necessary to achieve a reliability of 0.80, whereas six monitoring days were needed to estimate reliable measures of MVPA. Between 38–57%, 55–73%, 65–80%, 71–84%, 76–87%, 79–89%, and 81–90% of the variance was accounted for using 1 to 7 days monitoring to represent habitual activity for overall PA and MVPA, respectively.

Table 3 presented the reliability coefficient associated with different number of monitored days stratified by sociodemographic characteristics and body mass. In terms of overall PA, a minimum of four days of monitoring show Intra-class reliability coefficient values ≥0.80 for all groups of skin color, socioeconomic position, job characteristics, and pre-pregnancy BMI, except for pregnant women with age ≥ 40 years. Reaching intraclass reliability coefficient values ≥0.80 required a minimum of seven days of use for MVPA, except for pregnant women at extremes of age and with non-white skin color.

**Table 3:** Intraclass reliability correlation coefficient for overall PA and MVPA stratified by maternal age, SES and paid job during pregnancy in pregnant women belonging to the 2015 Pelotas (Brazil) Birth Cohort Study.

Intraclass reliability coefficient using the Spearman-Brown Prophecy Formula														
	Overall PA							MVPA^a^						
	1 day	2 days	3 days	4 days	5 days	6 days	7 days	1 day	2 days	3 days	4 days	5 days	6 days	7 days
**Maternal age (years)**														
<20	0.52	0.69	0.77	0.81	0.85	0.87	0.88	0.30	0.46	0.56	0.63	0.68	0.72	0.75
20–29	0.57	0.72	0.80	0.84	0.87	0.89	0.90	0.38	0.55	0.65	0.71	0.76	0.79	0.81
30–39	0.61	0.76	0.82	0.86	0.89	0.90	0.92	0.40	0.57	0.67	0.73	0.77	0.80	0.82
≥ 40	0.44	0.61	0.70	0.76	0.80	0.82	0.85	0.33	0.50	0.60	0.67	0.72	0.75	0.78
**Skin color**														
White	0.58	0.73	0.80	0.85	0.87	0.89	0.91	0.39	0.56	0.65	0.72	0.76	0.79	0.82
Black	0.57	0.73	0.80	0.84	0.87	0.89	0.90	0.36	0.52	0.62	0.69	0.73	0.77	0.79
Brown/Yellow/Indigenous	0.55	0.71	0.78	0.83	0.86	0.88	0.89	0.34	0.51	0.60	0.67	0.72	0.75	0.78
**SES (quintiles)**														
Q1(poorest)	0.59	0.74	0.81	0.85	0.88	0.90	0.91	0.39	0.56	0.66	0.72	0.76	0.79	0.82
Q2	0.53	0.69	0.77	0.82	0.85	0.87	0.89	0.35	0.52	0.62	0.68	0.73	0.76	0.79
Q3	0.60	0.75	0.82	0.86	0.88	0.90	0.91	0.34	0.50	0.60	0.67	0.72	0.75	0.78
Q4	0.55	0.71	0.79	0.83	0.86	0.88	0.89	0.36	0.53	0.63	0.70	0.74	0.78	0.80
Q5 (wealthiest)	0.56	0.72	0.80	0.84	0.87	0.89	0.90	0.37	0.54	0.64	0.70	0.75	0.78	0.80
**Paid job during pregnancy**														
No	0.58	0.73	0.80	0.84	0.87	0.89	0.90	0.37	0.54	0.63	0.70	0.74	0.78	0.80
Yes	0.57	0.72	0.80	0.84	0.87	0.89	0.90	0.40	0.58	0.67	0.73	0.77	0.80	0.83
**Pre-pregnancy BMI (kg/m**^**2**^**)**														
Underweight	0.57	0.73	0.80	0.84	0.87	0.89	0.90	0.36	0.53	0.63	0.69	0.74	0.77	0.80
Normal	0.58	0.73	0.80	0.85	0.87	0.89	0.91	0.40	0.57	0.67	0.73	0.77	0.80	0.82
Overweight	0.58	0.71	0.81	0.85	0.88	0.89	0.91	0.38	0.56	0.65	0.71	0.76	0.79	0.81
Obese	0.55	0.71	0.78	0.83	0.86	0.88	0.89	0.39	0.56	0.65	0.71	0.76	0.79	0.81

*SES: socioeconomic position; MVPA: moderate-to-vigorous physical activity; PA: physical activity; BMI: body mass index.

^a^ analyses were performed using log-transformed MVPA

**Fig 2:**
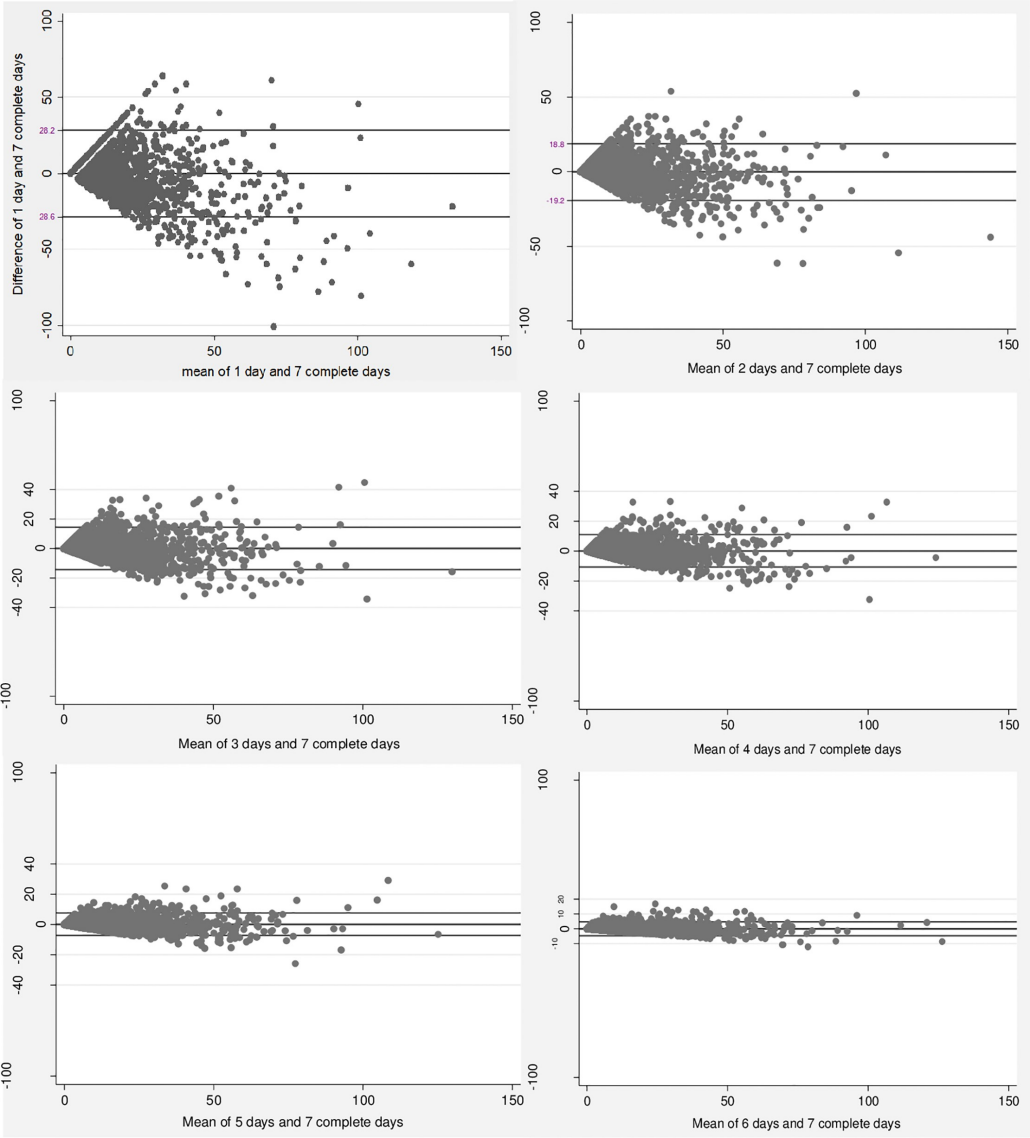
Bland-Altman plots of the comparison between the means of measurement days (1 to 6) and the standard of seven complete days of measurement for moderate-to-vigorous physical activity.

**Fig 3:**
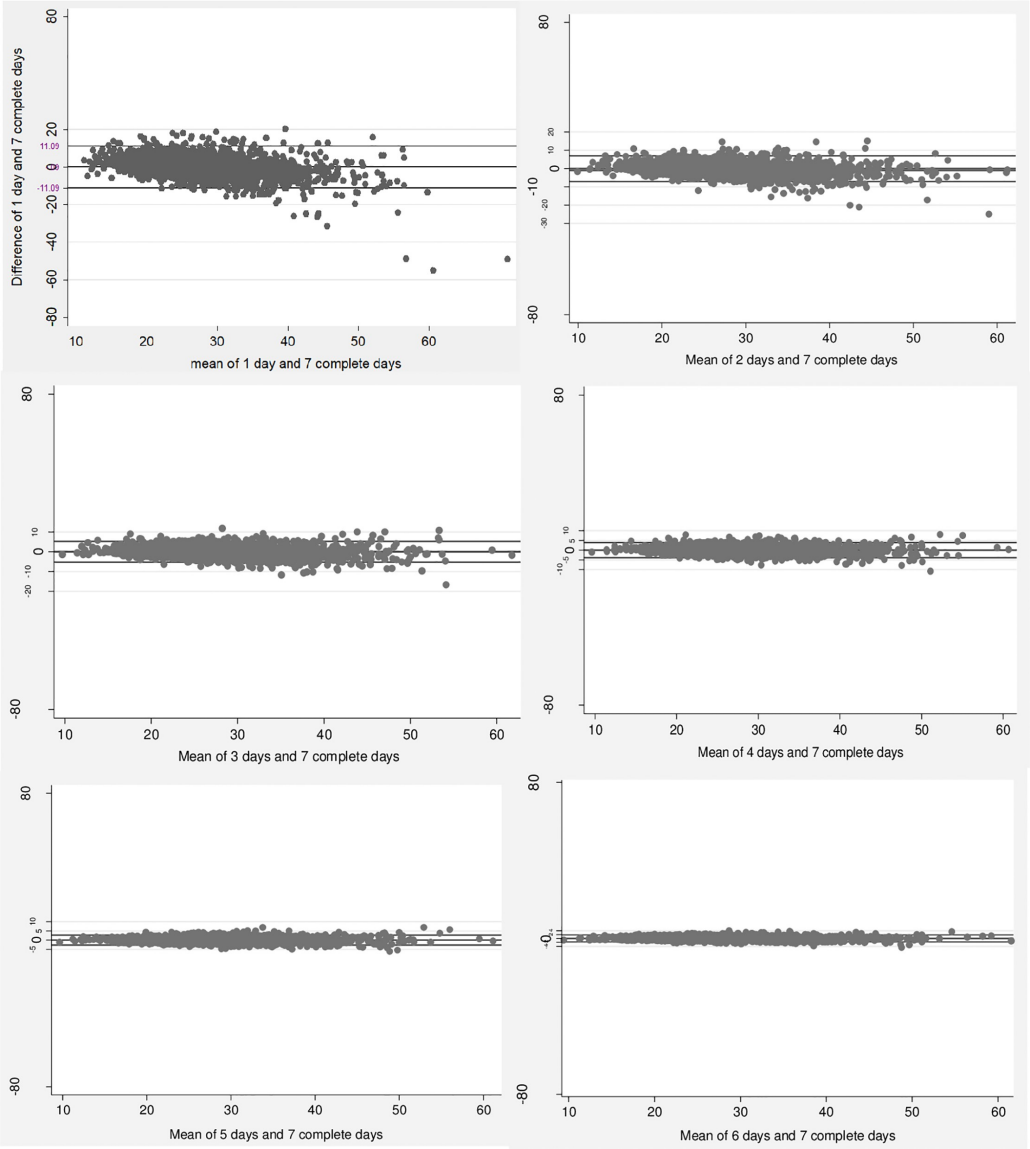
Bland-Altman plots of the comparison between the means of measurement days (1 to 6) and the standard of seven complete days of measurement for overall physical activity.

Bland-Altman plots indicated on average differences between number of days near zero, narrow limits of agreement, and homogeneous variability across the days of monitoring for both overall PA and MVPA. More days of monitoring produced lower variability between measurement days (1 to 6) and the standard seven-day protocol for both MVPA and overall PA (**Figs 2 and 3**).

Higher mean differences were found between one day and seven complete days for both MVPA (mean difference: 0.36; 95% CI: -0.31–1.02) and overall PA (mean difference: 0.09; 95% CI: -0.15; 0.33). On the other hand, lower mean differences were identified between six days of measurement and the standard protocol in the two intensities investigated, MVPA (mean difference: -0.11; 95%CI: -0.21; -0.01) and overall PA (mean difference: -0.03; 95%CI: -0.06; 0.01), respectively.

## Discussion

This study determined the number of monitoring days needed to obtain reliable estimates of overall PA and MVPA in pregnant women using wrist-worn accelerometers in a population-based study in southern Brazil. Our findings showed that seven monitoring days of the week should be considered to achieve a reliability of at least 0.80 to accurately predict both overall PA and MVPA. Variability in the means of overall PA and MVPA across the days of the week was also observed, with the lowest means of overall PA and MVPA on Sunday. This finding indicates that weekend days cannot be ignored in the design and analysis of PA studies. Considered together, these findings support the usual approach of asking adults to wear an accelerometer for one week.

To the best of our knowledge, this is the first study to date to investigate the number of days needed to obtain reliable estimates of overall PA and MVPA during pregnancy in a representative population sample using raw triaxial wrist accelerometry. Literature in other populations indicate that the number of days needed to obtain reliable PA estimates varies according to PA intensity. A study conducted by Dillon et al. [12], using wrist-worn GENEActivb accelerometers investigated an acceptable reliability measure of weekly habitual PA in middle-aged Irish adults. They also found that the monitoring frame duration for reliable estimates varied across PA intensity. Results ranged from 2 days when evaluating combined MVPA to 6 days for specifically vigorous activities. Matthews et al. [26] using the Computer Science Applications (CSA) accelerometer on the hip in healthy adults determined that 3–4 days monitoring were required to accurately measure MVPA. Similar results were reported by Hart et al. [13] in a study with older adults using hip-worn accelerometers. Contrary to these findings, we observed that six monitoring days are necessary to produce reliable measures of MVPA among pregnant women. Pregnancy is a period that involves many physical and psychological changes including morphological adjustments for fetal development, changes in mood, anxiety, and fatigue/energy [27]. These factors may contribute to a larger variability in MVPA measurements throughout the week in pregnant women compared to other populations.

PSeveral aspects may explain the inconsistency in the number of days required for reliable PA assessment, such as the heterogeneity in the type of accelerometer adopted, number of accelerometers worn, and placement of the device (hip or wrist). Another difference is the statistical techniques applied to obtain stable mean estimates of PA. The discrepancies in methods across studies emphasize the need to establish an appropriate monitoring frame to reliably capture habitual physical behavior for each population, accelerometer, PA intensity, and body position in the device is worn [28].

Patterns of PA during pregnancy are influenced by sociodemographic, health, environmental, and behavioral characteristics [15, 28]. Considering the possible influence of these aspects on the number of days required to represent weekly habitual PA, analyses were explored by these characteristics. Similar results were found for all groups except for pregnant women younger than 20 years, who needed more than 7 days of monitoring to achieve reliable measures of MVPA.

The valid and reliable accelerometer, 24-hour study protocol, large sample size, high-rate response rate, wrist-worn accelerometer, and statistical techniques employed are strengths of our study. However, some limitations should be noted. The cut point applied (> 100 mg) may not be an appropriate threshold to determine MVPA during pregnancy. However, we used MVPA >100 mg because there are no specific cut points validated for pregnant women using raw data placement on the wrist. Also, wrist-worn compared to hip-worn accelerometry generally improves participant compliance [1], and previous studies have used the same methodological approach [21,22].

In our study, accelerometers were used for seven complete, consecutive days and only during the 2^nd^ trimester of pregnancy. Monitoring for longer periods, such as a month, season or a year, would provide greater representativeness of habitual PA behavior, particularly given that many studies have reported seasonal and monthly variations in PA [29, 30]. However, a longer period of data collection would probably result in lower compliance and bring logistic issues during collection (such as battery replacement and data downloading). Also, our results showed that measuring seven consecutive days could reliably estimate overall PA and MVPA in this group of pregnant women.

An important question is the number of accelerometer monitoring days needed to obtain a stable group-level mean estimate of PA measured over a week. Results by Wolff-Hughes et al. [10] suggested that stable estimates of group-level PA can be obtained from as little as one randomly selected day of monitoring from a sampled week. It is important to clarify that the research question and statistical techniques applied were different from our study, since in contrast we were interested in addressing the optimal number of days needed to obtain reliable estimates of overall PA and MVPA.

In addition, our findings are not advocating for future studies among pregnant using only three (to estimate overall PA) or seven monitoring days (to estimate MVPA).This study suggests that a seven day protocol may be optimal when assessing habitual PA in pregnant women. If a shorter time of assessment is applied, there will be no room for addressing non-wear time, which might lead to a larger loss due to compliance criteria.

## Conclusion

Our results indicated that among pregnant women in the 2^nd^ trimester of pregnancy at least three days of monitoring are required to reliably capture overall PA and seven days monitoring when considering MVPA. Due to the substantially lower PA levels during Sundays, we recommend a seven consecutive day protocol when assessing habitual PA in the 2nd trimester of pregnancy. These findings may have implications for future study designs and data reduction strategies among accelerometer-assessed physical activity studies of pregnant women.
